# Supplementary material for: The rise and global spread of IMP carbapenemases (1996-2023): a genomic epidemiology study
Source: Nat Commun. 2025 Dec 9;17:183. doi: 10.1038/s41467-025-66874-7 (PMC12780205; doi:10.1038/s41467-025-66874-7)
Supplement: Supplementary file 1 — Supplementary Information [file 41467_2025_66874_MOESM1_ESM.pdf]

Supplementary information for: The rise and global spread of IMP carbapenemases (1996-2023): a genomic epidemiology study

Authors

Ben Vezina[1+], Bhargava Reddy Morampalli[1+], Hoai-An Nguyen[1], Angela Gomez-Simmonds[2], Anton Y. Peleg[1,3,4], Nenad Macesic[*1,3,5]

[1] Department of Infectious Diseases, The Alfred Hospital and School of Translational Medicine, Monash University, Melbourne, Australia

[2] Division of Infectious Diseases, Department of Internal Medicine, UC Davis Health, Sacramento, California, USA

[3] Centre to Impact AMR, Monash University, Clayton, Australia

[4] Infection Program, Monash Biomedicine Discovery Institute, Department of Microbiology, Monash University, Clayton, Australia

[5] Infection Prevention & Healthcare Epidemiology, Alfred Health, Melbourne, Australia.

[+] These authors contributed equally

* Corresponding Author Dr. Nenad Macesic, Department of Infectious Diseases, The Alfred Hospital and School of Translational Medicine, Monash University, Level 1 Alfred Lane House, Alfred Hospital, 55 Commercial Rd, Melbourne, VIC 3004, Australia. Email: nenad.macesic1@monash.edu

Supplemental Figures

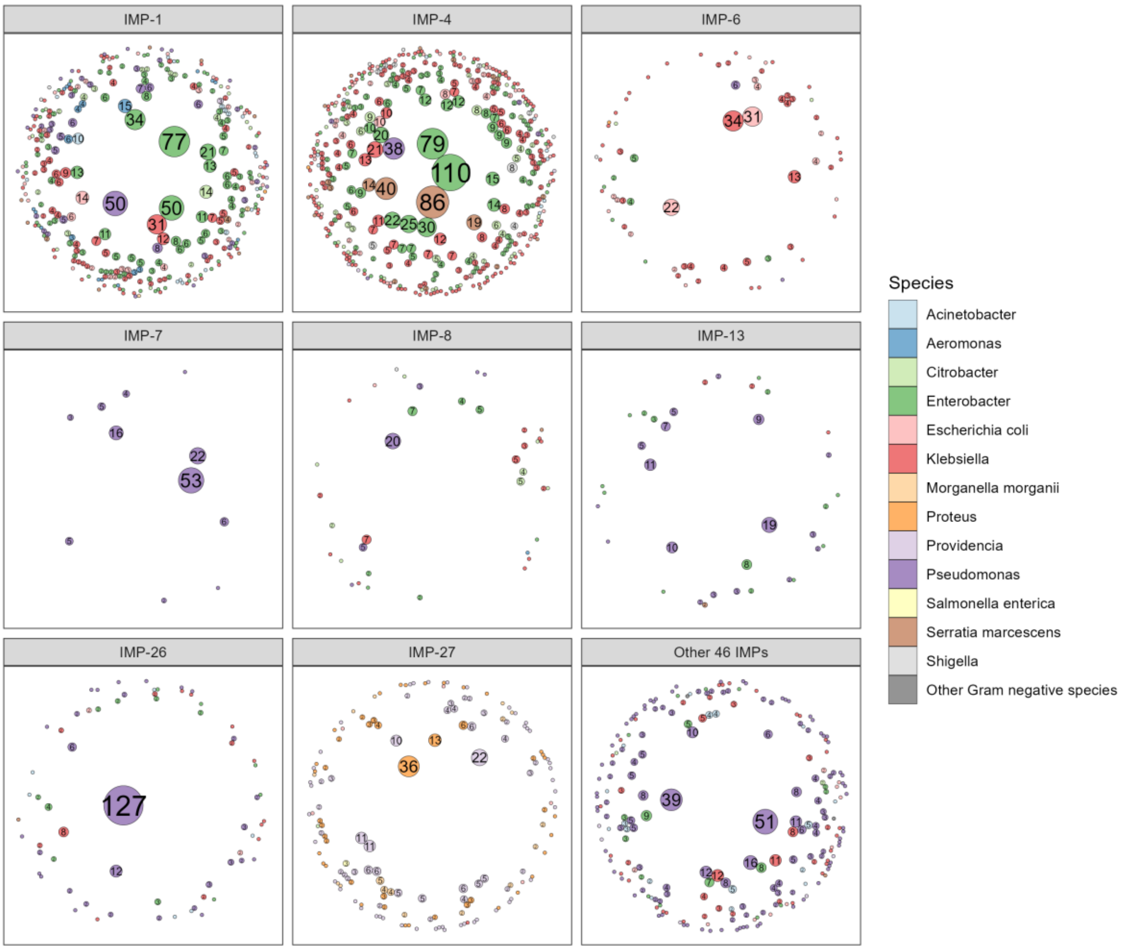

Fig. S1: Size of IMP-clusters within dataset. Number of genomes within IMP-cluster shown by size of bubble and text. No text is shown for IMP-clusters with n=1 genome.

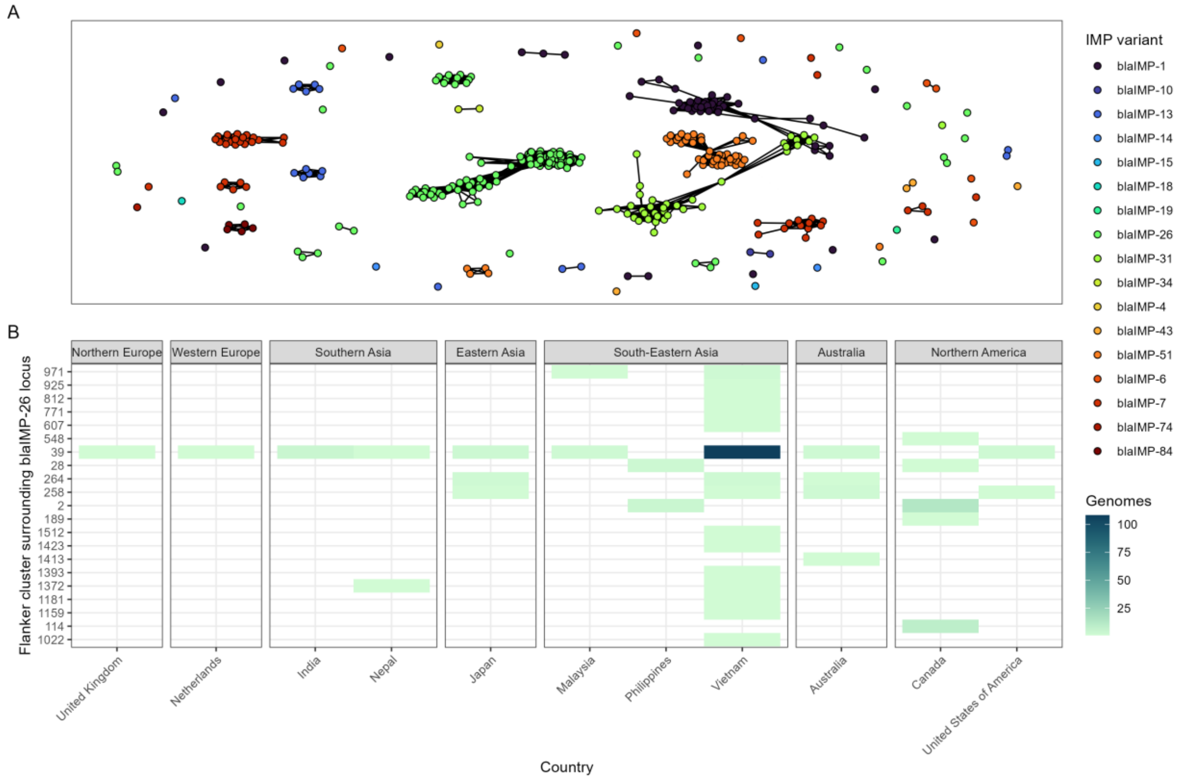

Fig. S2: *bla*$_{\text{IMP}}$-carrying *P. aeruginosa* ST235 has global and diverse spread **A:** Network of clonally-linked *P. aeruginosa* ST235 genomes, coloured by *bla*$_{\text{IMP}}$ variant. **B:** Heatmap of IMP-26-containing *P. aeruginosa* ST235 showing regional-specific independent acquisition of the IMP-26 gene.

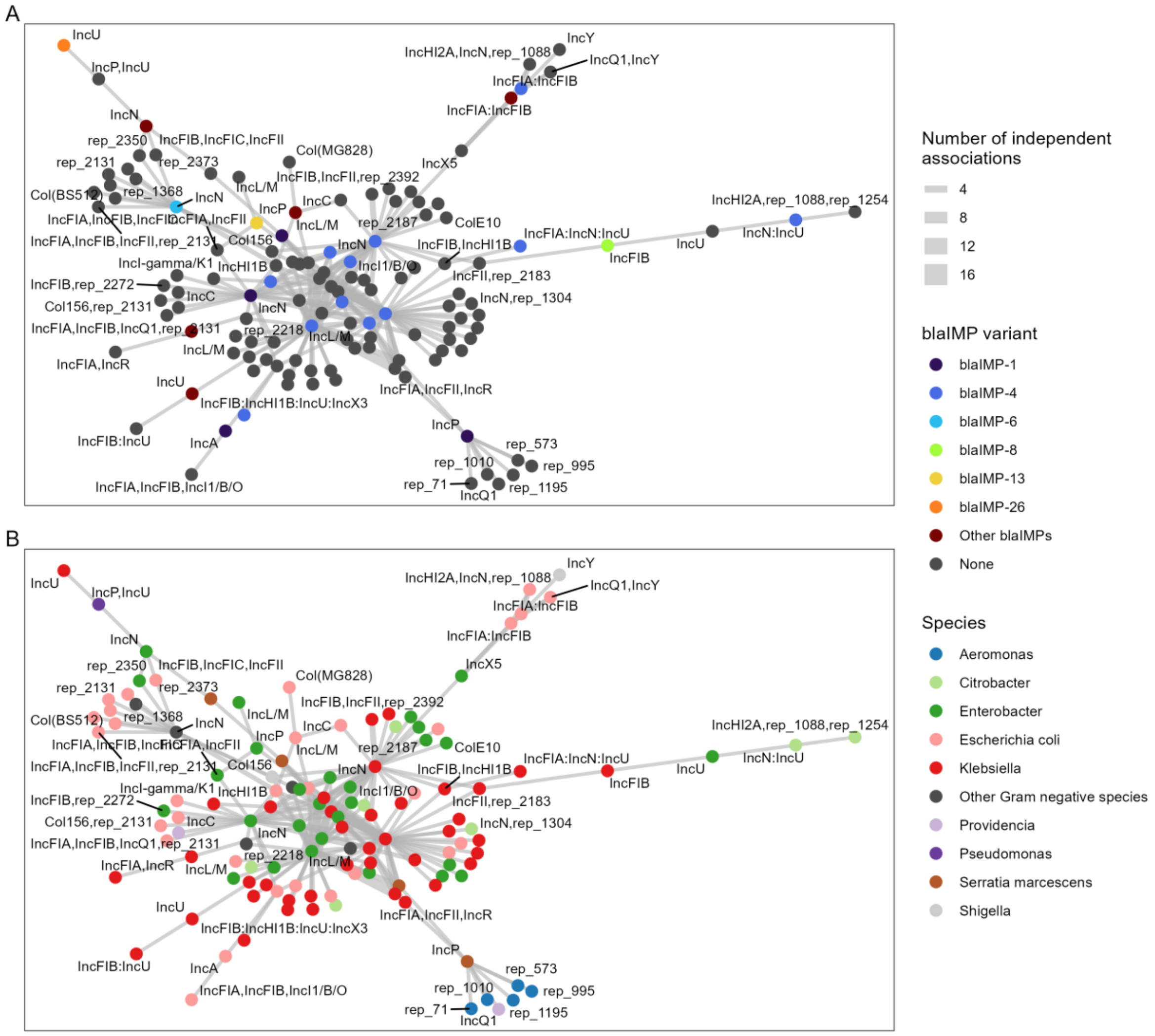

Fig. S3: Network of co-occurring *bla*$_{IMP}$-plasmids to non-*bla*$_{IMP}$ plasmids within genomes.

Genomes were dereplicated into IMP-clusters prior to analysis to remove clonal bias. Links are drawn between plasmids if they co-occur within genomes, while the thickness of the links show the number of genomic co-occurrences. **A:** Network coloured by IMP variant. **B:** Network coloured by species.

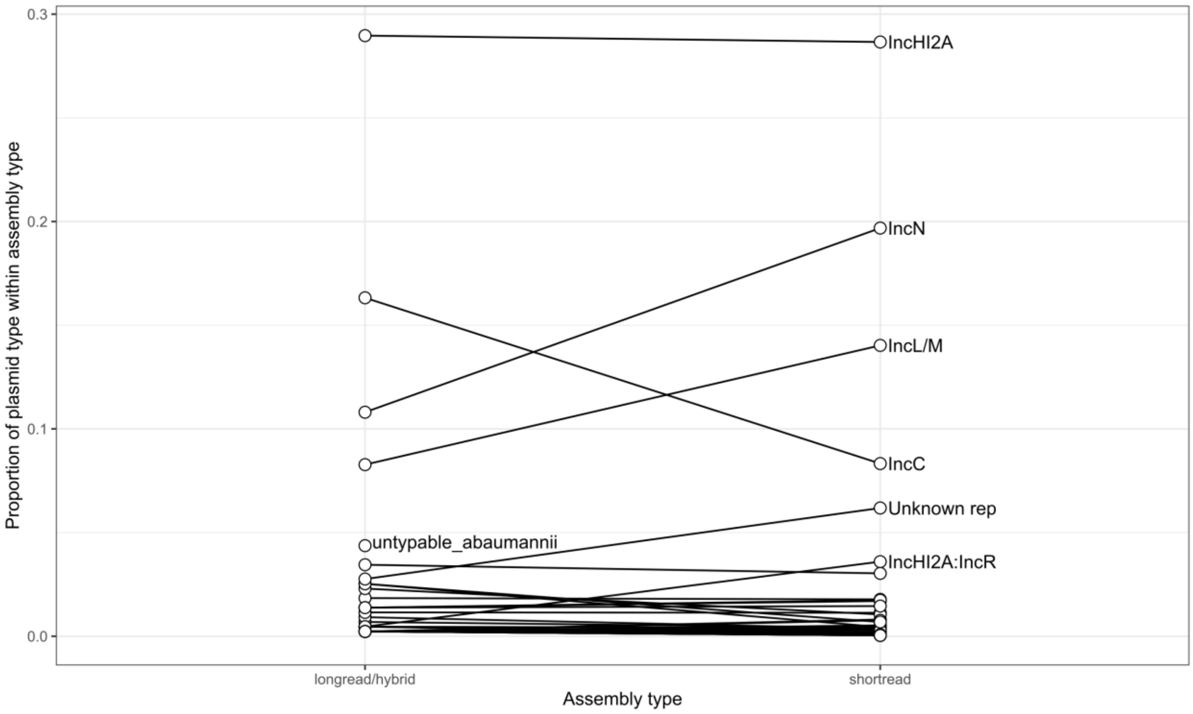

Fig. S4: Proportion of plasmid clusters in long-read/hybrid dataset vs full dataset including short read draft assemblies.

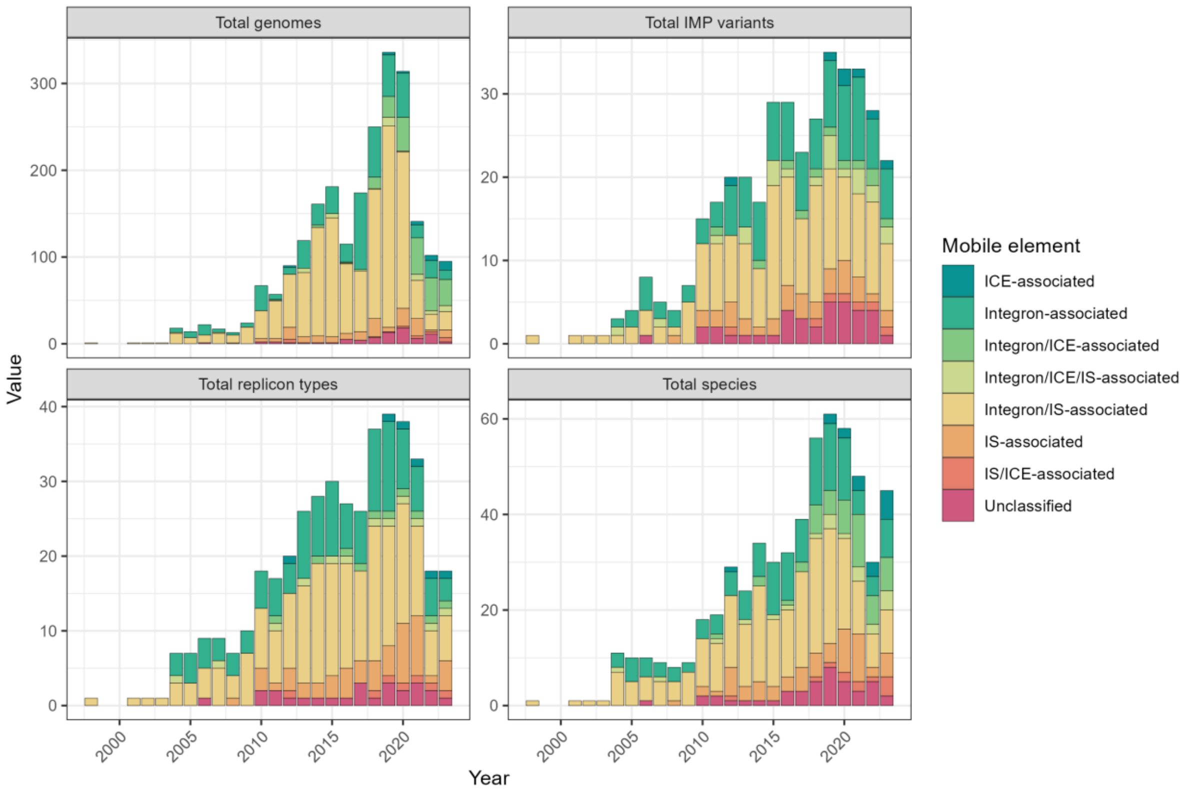

Fig. S5: Mobile elements have been associated with IMP carbapenemases over time

Cumulative column graph showing the breakdown of associated mobile elements and their spread across numbers of species, *bla*$_{IMP}$ variants, total genomes and plasmid types. Raw data found in Table S1.

Supplementary data legends

Supplementary data 1: Table of all data used in this study, including accession numbers, analysis results and metadata.

Supplementary data 2: bla_{IMP} variants over time, supporting information for Fig. 1.

Supplementary data 3: bla_{IMP-26} and bla_{IMP-27} supporting information for Fig. 3.

Supplementary data 4: bla_{IMP} variants and their global distributions

Supplementary data 5: Association between bacterial lineages and bla_{IMP} variants

Supplementary data 6: IMP-clusters and their global distribution and makeup

Supplementary data 7: Plasmid clusters and their spread across multiple countries and geographical regions for long read only genomes

Supplementary data 8: 'Propagator' strain-plasmid pairings and 'connector' strains.

Supplementary data 9: plDDT scores as predicted by AlphaFold2 and Colabfold

Supplementary data 10: Summary of isolation sources and genome counts, supporting information for Fig. 7

Supplementary data 11: Summary of plasmids moving between isolation sources and ≥2 species

Source data legends

Source data Fig 1B: Source data for Fig. 1B

Source data Fig 1C: Source data for Fig. 1C

Source data Fig 2BD: Source data for Fig. 2BD

Source data Fig 7B: Source data for Fig. 7B
