# Supplementary material for: The rise and global spread of IMP carbapenemases (1996-2023): a genomic epidemiology study
Source: Nat Commun. 2025 Dec 9;17:183. doi: 10.1038/s41467-025-66874-7 (PMC12780205; doi:10.1038/s41467-025-66874-7)
Supplement: Supplementary file 3 — Description of Additional Supplementary Files [file 41467_2025_66874_MOESM3_ESM.pdf]

Description of Additional Supplementary Files

File Name: Supplementary Data 1
Description: Table of all data used in this study, including accession numbers, analysis results and metadata.

File Name: Supplementary Data 2
Description: bla_{IMP} variants over time, supporting information for Fig. 1.

File Name: Supplementary Data 3
Description: bla_{IMP}-26 and bla_{IMP-27} supporting information for Fig. 3.

File Name: Supplementary Data 4
Description: bla_{IMP} variants and their global distributions

File Name: Supplementary Data 5
Description: Association between bacterial lineages and bla_{IMP} variants

File Name: Supplementary Data 6
Description: IMP-clusters and their global distribution and makeup

File Name: Supplementary Data 7
Description: Plasmid clusters and their spread across multiple countries and geographical regions for long read only genomes

File Name: Supplementary Data 8
Description: 'Propagator' strain-plasmid pairings and 'connector' strains.

File Name: Supplementary Data 9
Description: plDDT scores as predicted by AlphaFold2 and Colabfold

File Name: Supplementary Data 10
Description: Summary of isolation sources and genome counts, supporting information for Fig. 7

File Name: Supplementary Data 11
Description: Summary of plasmids moving between isolation sources and ≥2 species
