# Supplementary material for: The rise and global spread of IMP carbapenemases (1996-2023): a genomic epidemiology study
Source: Nat Commun. 2025 Dec 9;17:183. doi: 10.1038/s41467-025-66874-7 (PMC12780205; doi:10.1038/s41467-025-66874-7)
Supplement: Supplementary file 5 — Reporting Summary [file 41467_2025_66874_MOESM5_ESM.pdf]

nature portfolio

Corresponding author(s): Dr. Nenad Macesic

Last updated by author(s): Oct 27, 2025

Reporting Summary

Nature Portfolio wishes to improve the reproducibility of the work that we publish. This form provides structure for consistency and transparency in reporting. For further information on Nature Portfolio policies, see our Editorial Policies and the Editorial Policy Checklist.

Statistics

For all statistical analyses, confirm that the following items are present in the figure legend, table legend, main text, or Methods section.

n/a	Confirmed	
☐	☒	The exact sample size (*n*) for each experimental group/condition, given as a discrete number and unit of measurement
☒	☐	A statement on whether measurements were taken from distinct samples or whether the same sample was measured repeatedly
☒	☐	The statistical test(s) used AND whether they are one- or two-sided<br>*Only common tests should be described solely by name; describe more complex techniques in the Methods section.*
☒	☐	A description of all covariates tested
☒	☐	A description of any assumptions or corrections, such as tests of normality and adjustment for multiple comparisons
☒	☐	A full description of the statistical parameters including central tendency (e.g. means) or other basic estimates (e.g. regression coefficient) AND variation (e.g. standard deviation) or associated estimates of uncertainty (e.g. confidence intervals)
☒	☐	For null hypothesis testing, the test statistic (e.g. *F*, *t*, *r*) with confidence intervals, effect sizes, degrees of freedom and *P* value noted<br>*Give P values as exact values whenever suitable.*
☒	☐	For Bayesian analysis, information on the choice of priors and Markov chain Monte Carlo settings
☒	☐	For hierarchical and complex designs, identification of the appropriate level for tests and full reporting of outcomes
☒	☐	Estimates of effect sizes (e.g. Cohen's *d*, Pearson's *r*), indicating how they were calculated

Our web collection on statistics for biologists contains articles on many of the points above.

Software and code

Policy information about availability of computer code

Data collection	Public data was collected from the NCBI Pathogen Isolate Browser, NCBI GenBank and the Sequence Read Archive on 1/10/2023.
Data analysis	The following software was used: BLAST v2.15, Prokka v1.14.6, AMRFinderPlus v3.12.8 , mlst v.2.19.0 , PopPUNK v4.2.0 , MASH v2.3 , SKA v1.0.0, MOB-suite v3.1.8 , Flanker v0.1.5 , ISEScan v1.7.2.3 , ICEberg 2.0, minimap2 v2.26 , Integron_finder v2.0.5, SignalP v6.0, Clustal Omega v1.2.4, bio3d v2.4-4, Colabfold v1.5.5, FoldMason v1.763a428, ChimeraX v1.8.<br>The following R packages were used: R v4.4.1 (79) and RStudio v2024.09.0 (80). The following R packages were used: tidyverse v2.0.0 (81), colorspace v2.1-1 (82), viridis v0.6.5 (83), ggh4x v0.2.8 (84), ggstream v0.1.0 (85), maps v3.4.2 (86), scatterpie 0.2.4 (87), sf v 1.0-17 (88), rnaturalearth v1.0.1 (89), ggnewscale v0.5.0 (90), treemapify v2.5.6 (91), patchwork v1.3.0 (92), igraph v2.0.3 (65), qgraph v1.9.8 (93), ggraph v2.2.1 (94), ggforce v0.5.0 (95), ggalluvial v0.12.5 (96), ggtree v3.12.0 (97), treeio v1.28.0 (98) and aplot v0.2.3 (99).<br>Custom scripts used include ChimeraX scripLife Sciences Study Design<br>Life Sciences Study Designts at https://github.com/bananabenana/ChimeraX_scripts, TreeBranchLabeller at https://github.com/bananabenana/TreeBranchLabeller and all R code can be found at Figshare: https://doi.org/10.6084/m9.figshare.28440992

For manuscripts utilizing custom algorithms or software that are central to the research but not yet described in published literature, software must be made available to editors and reviewers. We strongly encourage code deposition in a community repository (e.g. GitHub). See the Nature Portfolio guidelines for submitting code & software for further information.

Data

Policy information about availability of data

All manuscripts must include a data availability statement. This statement should provide the following information, where applicable:

- Accession codes, unique identifiers, or web links for publicly available datasets
- A description of any restrictions on data availability
- For clinical datasets or third party data, please ensure that the statement adheres to our policy

All data used in this study is available as Supplemental Figs. S1-4 within the Supplementary Information document, Supplementary Data 1-11 and Source data. Additional supplemental data provided at Figshare: https://doi.org/10.6084/m9.figshare.28440992

Research involving human participants, their data, or biological material

Policy information about studies with human participants or human data. See also policy information about sex, gender (identity/presentation), and sexual orientation and race, ethnicity and racism.

Reporting on sex and gender	N/A
Reporting on race, ethnicity, or other socially relevant groupings	N/A
Population characteristics	N/A
Recruitment	N/A
Ethics oversight	N/A

Note that full information on the approval of the study protocol must also be provided in the manuscript.

Field-specific reporting

Please select the one below that is the best fit for your research. If you are not sure, read the appropriate sections before making your selection.

☒ Life sciences ☐ Behavioural & social sciences ☐ Ecological, evolutionary & environmental sciences

For a reference copy of the document with all sections, see nature.com/documents/nr-reporting-summary-flat.pdf

Life sciences study design

All studies must disclose on these points even when the disclosure is negative.

Sample size	Sample size of the dataset (n=4556 genomes) was determined by the number of blaIMP-positive genomes available public genome databases. As this manuscript analyses the evolutionary history of blaIMP, this is a sufficient and reasonable sample size.
Data exclusions	No data were excluded from the analyses
Replication	Both first-authors replicated each computational analysis. All attempts at replication were successful
Randomization	Not relevant to study - no intervention/control groups
Blinding	Not relevant to study - no intervention/control groups

Reporting for specific materials, systems and methods

We require information from authors about some types of materials, experimental systems and methods used in many studies. Here, indicate whether each material, system or method listed is relevant to your study. If you are not sure if a list item applies to your research, read the appropriate section before selecting a response.

Materials & experimental systems

n/a	Involved in the study
☒	☐ Antibodies
☒	☐ Eukaryotic cell lines
☒	☐ Palaeontology and archaeology
☒	☐ Animals and other organisms
☒	☐ Clinical data
☒	☐ Dual use research of concern
☒	☐ Plants

Methods

n/a	Involved in the study
☒	☐ ChIP-seq
☒	☐ Flow cytometry
☒	☐ MRI-based neuroimaging

Plants

Seed stocks	*Report on the source of all seed stocks or other plant material used. If applicable, state the seed stock centre and catalogue number. If plant specimens were collected from the field, describe the collection location, date and sampling procedures.*
Novel plant genotypes	*Describe the methods by which all novel plant genotypes were produced. This includes those generated by transgenic approaches, gene editing, chemical/radiation-based mutagenesis and hybridization. For transgenic lines, describe the transformation method, the number of independent lines analyzed and the generation upon which experiments were performed. For gene-edited lines, describe the editor used, the endogenous sequence targeted for editing, the targeting guide RNA sequence (if applicable) and how the editor was applied.*
Authentication	*Describe any authentication procedures for each seed stock used or novel genotype generated. Describe any experiments used to assess the effect of a mutation and, where applicable, how potential secondary effects (e.g. second site T-DNA insertions, mosiacism, off-target gene editing) were examined.*
